# No evidence of sickness behavior in immune‐challenged field crickets

**DOI:** 10.1002/ece3.6349

**Published:** 2020-06-01

**Authors:** Clint D. Kelly, Jules Mc Cabe Leroux

**Affiliations:** ^1^ Département des Sciences biologiques Université du Québec à Montréal Montreal QC Canada

**Keywords:** adipsia, anorexia, diet, fever, immunity, sex differences, sickness behavior

## Abstract

Sickness behavior is a taxonomically widespread coordinated set of behavioral changes that increases shelter‐seeking while reducing levels of general activity, as well as food (anorexia) and water (adipsia) consumption, when fighting infection by pathogens and disease. The leading hypothesis explaining such sickness‐related shifts in behavior is the energy conservation hypothesis. This hypothesis argues that sick (i.e., immune‐challenged) animals reduce energetic expenditure in order have more energy to fuel an immune response, which in some vertebrates, also includes producing an energetically expensive physiological fever. We experimentally tested the hypothesis that an immune challenge with lipopolysaccharide (LPS) will cause *Gryllus firmus* field crickets to reduce their activity, increase shelter use and avoid foods that interfere with an immune response (i.e., fat) while preferring a diet that fuels an immune response (i.e., protein). We found little evidence of sickness behavior in *Gryllus firmus* as immune‐challenged individuals did not reduce their activity or increase their shelter‐seeking. Neither did we observe changes in feeding or drinking behavior nor a preference for protein or avoidance of lipids. Males tended to use shelters less than females but no other behaviors differed between the sexes. The lack of sickness behavior in our study might reflect the fact that invertebrates do not possess energetically expensive physiological fever as part of their immune response. Therefore, there is little reason to conserve energy via reduced activity or increased shelter use when immune‐challenged.

## Graphical abstract

Sickness behavior is a well‐documented phenomenon in vertebrate animals; however, sickness behavior is less documented in invertebrates. We show that immune‐challenged crickets exhibit very little behavioral modification compared with control crickets.

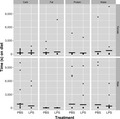

## 1 INTRODUCTION

Pathogens and disease are ubiquitous in nature and infection by them is an inevitability for animals. Consequently, natural selection has shaped animals to adaptively modify their behavior and physiology to maximize fitness under such conditions. In combination with behavioral (e.g., Adamo, 2008; Vaughn, Bernheim, & Kluger, 1974) or physiological (Roberts, 1991) fever, part of the adaptive response to infection is a coordinated set of behavioral changes that increases shelter‐seeking while reducing levels of social interaction, exploratory behavior, reproductive behavior, general activity, and food (anorexia) and water (adipsia) consumption (Ashley & Wingfield, 2012; Hart, 1988). This suite of adaptive behavioral changes is known as sickness behavior (Hart, 1988).

That sickness behavior can be induced by an immune challenge (e.g., lipopolysaccharide, LPS) in both vertebrates (Dantzer, 2004; see also Owen‐Ashley, Turner, Hahn, & Wingfield, 2006; Owen‐Ashley & Wingfield, 2006) and invertebrates (e.g., Adamo, Fidler, & Forestell, 2007; Bashir‐Tanoli & Tinsley, 2014; Dunn, Bohnert, & Russell, 1994; Kazlauskas, Klappenbach, Depino, & Locatelli, 2016) suggests that postinfection behavioral changes are due to a host response and not due to manipulation by the pathogen or the by‐products of infection (see also Johnson, 2002). The proximate mechanism underlying behavioral modifications involves interactions between nonspecific, innate immune responses, and the nervous and endocrine systems with mediation by proinflammatory cytokines (e.g., IL‐6) in vertebrates (Ashley & Wingfield, 2012; Dantzer & Kelley, 1989; Dantzer, O'Connor, Freund, Johnson, & Kelley, 2008; Hart, 1988; Johnson, 2002) and insects (Adamo, 2008, 2012; Ishii, Hamamoto, & Sekimizu, 2015).

Sickness behavior is not only observed across a wide range of animal taxa (reviewed in Sullivan, Fairn, & Adamo, 2016) including mammals (e.g., Bilbo, Drazen, Quan, He, & Nelson, 2002; Carlton & Demas, 2014; Hart, 1988), amphibians (e.g., Llewellyn, Brown, Thompson, & Shine, 2011), birds (e.g., Lopes, Adelman, Wingfield, & Bentley, 2012; Owen‐Ashley et al., 2006; Owen‐Ashley & Wingfield, 2006), and insects (e.g., Adamo, Bartlett, Le, Spencer, & Sullivan, 2010; Adamo et al., 2007; Ayres & Schneider, 2009; Bashir‐Tanoli & Tinsley, 2014; Bos, Lefèvre, Jensen, & d'Ettorre, 2012; Dunn et al., 1994; Kazlauskas et al., 2016; Sullivan et al., 2016) but the behaviors are remarkably similar across phyla despite taxa having markedly different physiologies and immune systems. Such phylogenetic conservatism surely attests to sickness behavior having fitness‐value, but what is its value? A leading hypothesis argues that sickness behavior adaptively functions to conserve energy because these behaviors permit the reallocation of energy to immunological defences including the production of fever (Ashley & Wingfield, 2012; Hart, 1988). Another hypothesis argues that sickness behavior reduces predation risk because these behaviors minimize exposure to predators through reduced activity and increased shelter use (Ashley & Wingfield, 2012; Dantzer, 2004; Dantzer & Kelley, 2007).

Though sickness behavior is generally consistent across animal taxa, within taxa, however, males and females can differ in the expression of sickness behaviors due to different life‐history demands and the timing of such demands. For example, mating behavior is inhibited in female, but not male, rats after administration of Il‐1 while the suppressive effects on activity are comparable in both sexes, thus suggesting that sex differences in sensitivity to IL‐1 is particular to sexual behavior (Yirmiya, Avitsur, Donchin, & Cohen, 1995). Inhibition of sexual behavior in female rats is likely adaptive because it prevents conception while the animal is sick, thereby minimizing the possibility of spontaneous abortion or abnormal development of offspring (Yirmiya et al., 1995). Males, on the other hand, continue to mate while sick because this strategy maximizes male fitness (Ashley & Wingfield, 2012). Sexual dimorphism might also arise because the sexes differ physiologically. For example, immune‐challenged male *Drosophila melanogaster* experience a greater downregulation of metabolic rate than females despite both sexes having similar food intakes (Bashir‐Tanoli & Tinsley, 2014). However, because females are able to resorb their eggs to use as metabolic fuel, they are able to maintain (or even increase) their metabolic rate while food acquisition is restricted during an immune response.

Restricted food and water intake are common responses by vertebrates (Ashley & Wingfield, 2012) and invertebrates (e.g., Bashir‐Tanoli & Tinsley, 2014; Sullivan et al., 2016) to infection. However, because physiological fever, and the activation and maintenance of immune responses require considerable energy to fuel, energy reserves will decline over time. This will require that at some point animals will need to resume feeding (if they have ceased) to replenish their energy reserves. The time until refeeding will likely depend on many factors including the condition of the animal; individuals in poor body condition are expected to resume feeding sooner after the onset of illness than an individual with greater energy stores (Ashley & Wingfield, 2012). In some cases, anorexic individuals might not completely cease food acquisition but rather might feed selectively (Kyriazakis, Tolkamp, & Hutchings, 1998) to avoid, for example, fat because dietary lipid can reduce immune function (Adamo et al., 2010). Protein might also be avoided because it generally contains micronutrients, such as iron, zinc, and copper, that are limiting for bacterial growth (Aubert, Goodall, & Dantzer, 1995; reviewed in Ashley & Wingfield, 2012). Some studies in insects have indeed shown that immune‐challenged individuals prefer carbohydrate‐rich diets compared with protein‐rich ones (Graham et al., 2014; Ponton et al., 2020). On the other hand, if mounting an immune response increases demands for protein then immune‐challenged animals should seek protein‐rich diets (Lee, Cory, Wilson, Raubenheimer, & Simpson, 2006; Povey, Cotter, Simpson, Lee, & Wilson, 2009). There is little general consensus as to what type of macronutrient immune‐challenged animals should prefer.

In this study, we experimentally test the hypothesis that *Gryllus firmus* field crickets that are immune‐challenged with lipopolysaccharide (LPS) will adaptively exhibit sickness behaviors including increased shelter use, decreased locomotion, decreased activity, and decreased food and water intake. We expect immune‐challenged crickets to be anorexic (reduce food consumption compared with controls), but we also expect that when they do eat they will selectively consume protein rather than carbohydrate and fat. In addition, we address the more rarely tested prediction of whether the sexes differ in sickness behaviors.

## 2 METHODS

### 2.1 Experimental animal rearing

Experimental crickets were lab‐reared descendants from wild individuals caught near Gainsville, FL. Crickets were maintained in a growth chamber at a constant temperature (28°C) and humidity (60%) with 12‐hr day/night light schedule. We maintained colony animals in 70‐L mixed‐sex bins of about 50 adult individuals, provided with cotton‐plugged water vials and ad libitum Iams™ Proactive Health™ adult original cat food. Each bin was provisioned with stacked cardboard egg cartons to provide refuge and wire mesh lids to provide ventilation. Crickets were isolated individually in small deli cups prior to final eclosion to ensure virginity. Crickets were not fed 24 hr prior to testing to increase their motivation to feed during trials (Sullivan et al., 2016).

### 2.2 Immune challenge

Lipopolysaccharide (LPS) is a nonpathogenic and nonliving elicitor that stimulates several pathways in the immune system of insects (Ahmed, Baggott, Maingon, & Hurd, 2002; Kelly, 2011; Moret & Schmid‐Hempel, 2000) including gryllid crickets. For example, LPS causes a reduction in daily calling rate (*G. campestris*, Jacot, Scheuber, & Brinkhof, 2004), terminal investment by males (*G. texensis*, Kelly, Telemeco, & Bartholomay, 2015) and females (*Acheta domesticus*, Adamo, 1999), prolonged development to adulthood (*G. texensis*, Kelly, Tawes, & Worthington, 2014), the production of significantly smaller spermatophores (*Gryllodes sigillatus*, Kerr, Gershman, & Sakaluk, 2010), immune system activation (*G. firmus*, Park & Stanley, 2015; *A. domesticus*, Charles & Killian, 2015), and affects the expression of sexually selected traits (*G. campestris*, Jacot, Scheuber, Kurtz, & Brinkhof, 2005). Immediately prior to behavioral testing (see below), crickets (7–12 days posteclosion) were anesthetized by being placed inside a 50 ml tube on ice for 390 s. We then haphazardly assigned each to an immune status treatment and administered either a 5 µl injection of phosphate‐buffered saline (PBS; Sigma‐Aldrich; control) or 100 µg of LPS (Sigma‐Aldrich) derived from the bacterium *Serratia marcescens* dissolved in 5 µl of PBS (experimental). Park and Stanley (2015) found that 100 µg of LPS elicited a significant immune response (nodulation) within 1 hr after injection in adult *G. firmus* crickets. All injections were given into the hemocoel, through the membrane between the sixth and seventh abdominal sternites using a 10 µl Hamilton syringe equipped with a 26s‐gauge needle. Separate syringes were used for injecting saline and LPS. Syringes were rinsed with ethanol and distilled water between injections and injection sites on crickets were sterilized with an ethanol‐soaked cotton ball prior to injection.

### 2.3 Behavioral trials

We measured the postinjection behavior of crickets using Ethovision^®^ XT video tracking software (Noldus, Spink, & Tegelenbosch, 2001) and PhenoTyper^®^ observation arenas (30 × 30 × 35 cm), outfitted with a built‐in infrared camera for overhead behavioral recording. After injection, crickets were placed individually in an arena containing a shelter (a 59 ml inverted plastic cup) and four plastic dishes (35 mm diameter). Each dish contained 0.03 g of either protein (3:1:1 mix of casein, peptone, and albumen [42%], cellulose [56.2%], Wesson's salt mixture [1.8%]), carbohydrate (42% carbohydrate diet consisting of equal parts 1:1 mix of sucrose and dextrin [42%], cellulose [56.2%], Wesson's salt mixture [1.8%]), fat (organic, fresh‐pressed flax oil [Flora] [42%], cellulose [56.2%], Wesson's salt mixture [1.8%]), or water (a small water‐soaked cotton ball). The shelter was placed in the center of the arena and contained a single exit/entrance hole. The diet and water dishes were randomly assigned to a corner of the arena and placed 6 cm from the arena sides to avoid sampling bias due to thigmotaxis. We video‐recorded crickets for 3 hr to maximize behavioral observation time after immune activation while also maximizing the number of samples processed (~16 animals per day). In each trial, we recorded each cricket's distance traveled (cm), speed (cm/s), activity (movement not necessarily involving displacement of the center tracking point, e.g., a cricket turning “on the spot”), amount of time spent in the shelter (s), and on the three diets and water (s). We used time spent on the diets and water as a proxy for feeding duration and thus diet choice. The difference in food mass before and after a trial would provide an ideal measure of food consumption (and diet choice); however, we could not weigh the food after the trial because crickets tend to defecate into the food dish and removal of the feces is not possible without also removing some food. However, two lines of evidence support our experimental protocol of using time spent on each of the diets. First, pilot observations showed that crickets typically consumed the diet while in the dish. Second, crickets that visited (and presumably fed on) a diet at least once weighed significantly more at the end of a trial than those that did not visit a diet (did not visit: 0.67 ± 0.0021 g; visited: 0.68 ± 0.0011 g; ANCOVA controlling for pretrial body mass: *F* = 17.73, *df* = 1, 645, *p* < .0001).

We excluded *n* = 44 videos due to missing data as a result of tracking errors. Crickets were weighed (to the nearest 0.001 g) immediately before and after their trial using a Sartorius (Göttingen, Germany) analytical balance. We measured each cricket's pronotum length (mm) after each trial. Pronotum length, a proxy measure of structural body size (see Kelly et al., 2014), was defined as the distance between the anterior and posterior edges of the pronotum and was measured to the nearest 0.001 mm under a Leica S6D stereomicroscope using Leica Application Suite (LAS) image analysis software (Leica Microsystems Inc.). Trials were conducted in the dark and each cricket was used in one trial only.

### 2.4 Statistical analysis

We tested the assumption that individuals assigned to the PBS and sickness (i.e., immune‐challenged) treatments did not differ in age by using a Poisson regression because the response variable was a positive integer. Treatment differences in pronotum length or pretrial body mass were tested by using a general linear model for each sex separately because of sexual size dimorphism in this species (e.g., Wey, Réale, & Kelly, 2019).

We performed an ANCOVA to determine whether mass change during the trial was related to sex or treatment. We first conducted a heterogeneity of slopes test by entering post‐trial mass as the response variable, and sex, treatment, initial mass, and their interactions as independent factors into a general linear model. If the three‐ and two‐way interactions between sex, treatment, and pretrial mass were not statistically significant, they were removed and the ANCOVA was performed.

We tested the effect of sex, treatment, and their interaction on the frequency of shelter use, time in shelter, total distance traveled, speed, and activity by using separate general linear models. The time spent in the shelter and frequency of shelter use were analyzed using negative binomial models because data were zero‐inflated. Total distance traveled, speed, and activity were Box‐Cox transformed prior to analysis in order to meet the assumption that model residuals are normally distributed.

We quantified phenotypic correlations between all five recorded behaviors for each sex and treatment separately by using Pearson product‐moment correlations (*r*). *p*‐Values were adjusted for multiple tests using Holm's method.

We tested the effect of sex and treatment on the time spent on each diet and water by using a generalized linear mixed model with sex and treatment entered as fixed independent factors and cricket ID entered as a random effect. Cricket ID was entered as a random effect because each cricket contributed four data points to the dataset (one for each nutrient). Models testing visitation frequency used a Poisson error distribution because the response variable was a count whereas models testing time on diets and water used a Gaussian distribution. Full (all interactions included) and reduced models (interactions removed) were compared by using AIC and chi‐square tests using the *anova* function in R (R Development Core Team, 2013). Post hoc tests of sex and treatment effects on visitation to and time on each of the three diets and water were examined using the R package *emmeans* (Lenth, Singmann, & Love, 2019).

Means are given ±1 standard deviation unless otherwise noted. All analyses were conducted in the R (version 3.1.2) statistical environment (R Development Core Team, 2013).

## 3 RESULTS

### 3.1 Phenotypes of experimental crickets

As expected, the age (number of days posteclosion) of experimental crickets did not differ between the sexes or treatments (sex: estimate ± *SE* = −0.02 ± 0.082, *z* = −0.27, *df* = 1, 158, *p* = .78; treatment: estimate ± *SE* = 0.03 ± 0.09, *z* = 0.32, *df* = 1, 158, *p* = .75; sex × treatment interaction: estimate ± *SE* = −0.01 ± 0.11, *z* = −0.049, *df* = 1, 158, *p* = .96; Table 1).

**TABLE 1:** Mean (±*SD*) measurements of four phenotypic traits in experimental male and female *Gryllus firmus* field crickets

Trait	Females		Males	
	Saline	LPS	Saline	LPS
Age (days)	8.17 ± 1.23	7.94 ± 0.96	7.94 ± 1.01	7.76 ± 0.96
Pronotum length (mm)	6.49 ± 0.33	6.23 ± 0.47	6.16 ± 0.36	6.26 ± 0.38
Pretrial mass (g)	0.80 ± 0.16	0.69 ± 0.11	0.58 ± 0.09	0.61 ± 0.08
Post‐trial mass (g)	0.83 ± 0.16	0.72 ± 0.12	0.61 ± 0.10	0.63 ± 0.10

Note

Mass (g) was taken for each cricket immediately before and after its trial. Sample sizes are: female saline: *n* = 31; female LPS: *n* = 30; male saline: *n* = 50; male LPS: *n* = 51.

PBS‐ and LPS‐injected males did not differ in their average pronotum length (estimate ± *SE* = −1.22 ± 0.91, *t* = −1.34, *df* = 1, 99, *p* = .18) or average pretrial mass (estimate ± *SE* = −0.04 ± 0.031, *t* = −1.4, *df* = 1, 99, *p* = .16; Table 1). In contrast, saline‐injected females were, by chance, significantly larger, on average, than LPS‐injected females (pronotum length: estimate ± *SE* = 3.28 ± 1.26, *t* = 2.59, *df* = 1, 59, *p* = .012; pretrial mass: estimate ± *SE* = 0.14 ± 0.047, *t* = 2.9, *df* = 1, 59, *p* = .005; Table 1).

A heterogeneity of slopes test showed no significant three‐ (sex × treatment × pretrial mass on post‐trial mass: estimate ± *SE* = 0.01 ± 0.07, *t* = 0.16, *df* = 1, 154, *p* = .87) or two‐way (sex × treatment: estimate ± *SE* = −0.02 ± 0.014, *t* = −1.4, *df* = 1, 155, *p* = .16; pretrial mass × treatment: estimate ± *SE* = −0.07 ± 0.035, *t* = −1.9, *df* = 1, 155, *p* = .063; pretrial mass × sex: estimate ± *SE* = 0.07 ± 0.035, *t* = 1.9, *df* = 1, 155, *p* = .053) interactions; we therefore removed all interaction terms and performed an ANCOVA that statistically controlled for initial body mass. We found no effect of sex (ANCOVA: estimate ± *SE* = 0.01 ± 0.01, *t* = 1.00, *df* = 1, 158, *p* = .32) or treatment (estimate ± *SE* =0.00 ± 0.0057, *t* = −0.68, *df* = 1, 158, *p* = .5) on mass gain but, not surprisingly, pre and post‐trial mass were significantly correlated (estimate ± *SE* = 1.01 ± 0.02, *t* = 59.64, *df* = 1, 158, *p* < .0001; Table 1).

### 3.2 Effect of sex and treatment on behaviors

Contrary to prediction, we found very little effect of sex, treatment, or their interaction on any of our five recorded behaviors (Table 2). We found only that males visited shelters significantly less frequently, on average, than females (Table 3).

**TABLE 2:** Mean (±*SD*) measurements of five behavioral traits in experimental male and female *Gryllus firmus* field crickets

Behavior	Females				Males			
	Saline	*n*	LPS	*n*	Saline	*n*	LPS	*n*
Shelter time (s)	1,072.17 ± 1757.02	30	1,462.90 ± 2,108.33	31	898.07 ± 1,325.98	51	1,114.59 ± 1,520.29	50
Shelter visits	153.84 ± 483.56	30	78.37 ± 172.99	31	43.94 ± 62.00	51	48.96 ± 70.81	50
Distance (cm)	10,371.27 ± 9,608.12	30	12,262.33 ± 12,243.90	31	9,439.38 ± 8,272.48	51	11,429.48 ± 9,528.18	49
Speed (cm/s)	1.79 ± 2.22	30	1.58 ± 1.45	31	1.20 ± 0.79	51	1.55 ± 2.01	49
Activity	0.31 ± 1.10	30	0.11 ± 0.14	31	0.19 ± 0.64	51	0.59 ± 2.14	50

**TABLE 3:** Results from linear models testing the effect of sex and treatment on five behaviors in *Gryllus firmus* field crickets

Behavior	*N*	Predictor	*β*	*z*‐Value	*p*‐Value
Shelter time (s)	F: 61	Intercept	6.98 ± 0.32	21.82	.00
	M: 101	Sex (male)	−0.18 ± 0.41	−0.44	.66
		Treatment (saline)	0.31 ± 0.46	0.68	.50
		Interaction	−0.09 ± 0.58	−0.16	.87
Shelter visits	F: 61	Intercept	5.04 ± 0.28	17.84	.00
	M: 101	**Sex (male)**	**−1.25 ± 0.36**	**−3.48**	**.00**
		Treatment (saline)	−0.67 ± 0.40	−1.67	.09
		Interaction	0.78 ± 0.51	1.53	.13
Distance travelled (cm)	F: 61	Intercept	14.94 ± 0.71	21.06	.00
	M: 100	Sex (male)	0.31 ± 0.91	0.34	.73
		Treatment (saline)	0.97 ± 1.01	0.96	.34
		Interaction	−0.12 ± 1.28	−0.10	.92
Speed (cm/s)	F: 61	Intercept	0.98 ± 0.03	32.68	.00
	M: 100	Sex (male)	0.02 ± 0.04	0.44	.66
		Treatment (saline)	−0.02 ± 0.04	−0.40	.69
		Interaction	0.01 ± 0.05	0.12	.91
Activity	F: 61	Intercept	2.12 ± 0.11	19.93	.00
	M: 101	Sex (male)	−0.04 ± 0.14	−0.33	.74
		Treatment (saline)	−0.12 ± 0.15	−0.76	.45
		Interaction	−0.06 ± 0.19	−0.31	.76

Note

Time spent in shelter and frequency of shelter visits tested using negative binomial model. Data for distance traveled, speed, and activity were Box‐Cox transformed prior to analysis. Statistically significant main predictors are in bold.

### 3.3 Correlations between behaviors

PBS‐injected females and LPS‐injected males exhibited similar phenotypic behavioral correlations. We found that the time spent in a shelter by crickets positively correlated with the frequency of shelter visits in saline‐injected females (*r* = .57, *p* < .001) and LPS‐injected males (*r* = .54, *p* < .001; Table 4). Similarly, distance traveled positively correlated with average walking speed in saline‐injected females (*r* = .87, *p* < .001) and LPS‐injected males (*r* = .89, *p* < .001; Table 4).

**TABLE 4:** Phenotypic correlations (Pearson product‐moment coefficient, *r*) for all pairs of behaviors for each sex and treatment

Correlation	*r*	*p*‐Value
(a) Females: saline		
Time in shelter—shelter visits	**.57**	**.03**
Time in shelter—distance	−.30	1.00
Shelter visits—distance	−.12	1.00
Time in shelter—speed	−.35	1.00
Shelter visits—speed	−.15	1.00
Distance—speed	**.87**	**.00**
Time in shelter—activity	−.25	1.00
Shelter visits—activity	−.21	1.00
Distance—activity	.21	1.00
Speed—activity	.06	1.00
(b) Females: LPS		
Time in shelter—shelter visits	.09	1.00
Time in shelter—distance	−.18	1.00
Shelter visits—distance	.40	.86
Time in shelter—speed	−.15	1.00
Shelter visits—speed	.14	1.00
Distance—speed	.31	1.00
Time in shelter—activity	.29	1.00
Shelter visits—activity	−.05	1.00
Distance—activity	.21	1.00
Speed—activity	.13	1.00
(c) Males: saline		
Time in shelter—shelter visits	.22	1.00
Time in shelter—distance	−.30	.99
Shelter visits—distance	.09	1.00
Time in shelter—speed	−.23	1.00
Shelter visits—speed	.02	1.00
Distance—speed	.36	.32
Time in shelter—activity	−.15	1.00
Shelter visits—activity	.41	.09
Distance—activity	.44	.05
Speed—activity	.30	.92
(d) Males: LPS		
Time in shelter—shelter visits	**.54**	**.00**
Time in shelter—distance	−.09	1.00
Shelter visits—distance	.12	1.00
Time in shelter—speed	−.08	1.00
Shelter visits—speed	.14	1.00
Distance—speed	**.89**	**.00**
Time in shelter—activity	−.02	1.00
Shelter visits—activity	.21	1.00
Distance—activity	.38	.27
Speed—activity	.32	.84

Note

Statistically significant correlation coefficients after Holm's adjustment for multiple tests (*n* = 40) are in bold.

### 3.4 Time on diets and water

Significantly, more crickets sampled a diet and water at least once (*n* = 152) compared with never sampling a diet or water (*n* = 6; *χ* ^2^ = 274.09, *df* = 1, *p* < .001; Table 5). Approximately, half of all crickets (53%) visited each of the three diets and water during their trial (Table 5) and these crickets traveled significantly further during a trial than those visiting three or fewer dishes (*F* = 12.98, *df* = 1, 159, *p* < .001). We, therefore, restricted our analyses of time on each diet and water to only those individuals that sampled all four dishes during a trial (*n* = 86) in order to remove any bias due to crickets not aware of other available options.

**TABLE 5:** Proportion of the water and three diet dishes visited by PBS‐ and LPS‐injected male and female crickets during a 3 hr trial

Proportion (%)	Females		Males	
	Saline	LPS	Saline	LPS
0	1	1	2	2
25	2	2	5	5
50	3	4	5	4
75	8	8	10	14
100	16	16	29	25

For those crickets that visited a dish at least once, separate ordinary least‐squares linear regressions for each diet and water revealed that the time spent on a dish was significantly positively correlated with visitation frequency (carbohydrate: estimate = 10.25 ± 1.37, *F* = 7.49, *p* < .001; fat: estimate = 3.85 ± 0.81, *F* = 4.79, *p* < .001; protein: estimate = 32.10 ± 3.17, *F* = 10.14, *p* < .001; water: estimate = 4.85 ± 2.24, *F* = 2.16, *p* = .033).

**FIGURE 1:**
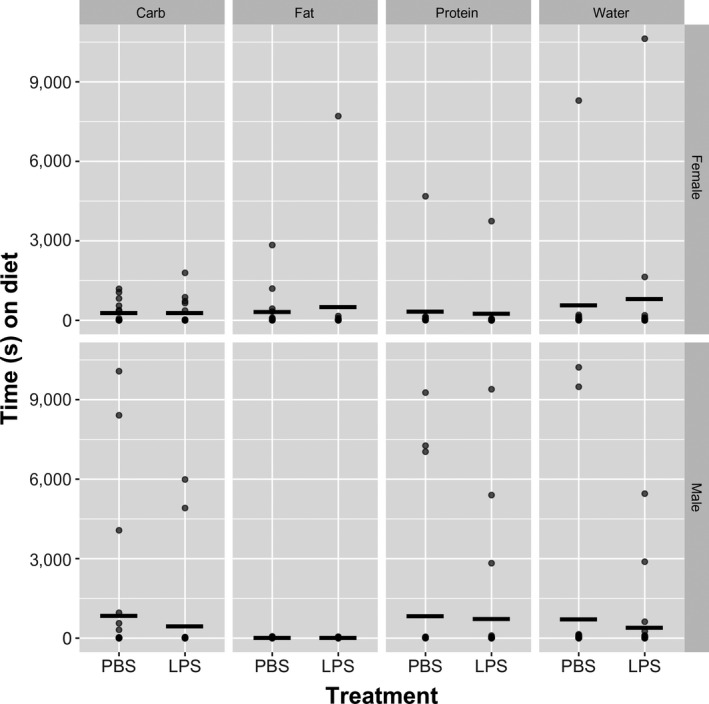
Duration (s) spent by female (*n* = 61) and male (*n* = 101) *Gryllus firmus* field crickets on water and each of three diets after injection with either saline or LPS. Dots represent individual crickets and horizontal bars represent the mean

For those crickets that sampled all four dishes during a trial, a reduced linear mixed model (cricket ID entered as a random effect) with the nonsignificant three‐way interaction removed was not a significantly better fit to the data than a full model (AIC_full_ = 3,853, AIC_reduced_ = 3,850; *χ* ^2^ = 2.31, *df* = 3, *p* = .51). The full model revealed a significant sex × diet interaction (*χ* ^2^ = 10.43, *df* = 3, *p* = .015), which suggests that LPS‐injection caused a significantly greater reduction in carbohydrate consumption in males than in females (Figure 1).

## 4 DISCUSSION

Sick animals are predicted to be more lethargic and risk‐averse (i.e., use shelters more often) compared with their healthy counterparts (Hart, 1988; Johnson, 2002). Our experimental study on sickness behavior in *Gryllus firmus* field crickets found little effect of sex, treatment, or their interaction on distance traveled, speed, activity, or duration of shelter use. We found only that males visited shelters significantly less frequently than females. This result is surprising since the burrow tends to play a significantly greater role in male versus female fitness in terms of mate attraction and mating success (Alexander, 1961). Our results, therefore, suggest that *G. firmus* crickets exhibit none of the classic sickness behaviors observed in other—particularly vertebrate—taxa. Moreover, our measured behaviors also failed to intercorrelate phenotypically within sex or treatment suggesting that there is little consistency among these behaviors (e.g., more active individuals do not travel further; slower individuals do not use shelters more).

We observed no reduction in activity‐related behaviors by immune‐challenged individuals, which is similar to Sullivan et al.'s (2016) finding in *G. texensis*. This general lack of behavioral modification by sick crickets could be due to the lack of physiological fever in crickets. Mammals are generally expected to reduce activity when infected to conserve their energy for physiological fever (Dantzer & Kelley, 2007). However, even in mammals in which heat conservation is not an issue (i.e., they live at lower latitudes), infected individuals are not expected to reduce activity.

In line with our findings, Sullivan et al. (2016) found that immune‐challenged *G. texensis* field crickets did not increase shelter use compared with control individuals. These authors offered a number of alternative explanations for these findings. One possibility is that immune‐challenged crickets remained outside of shelters to seek reproductive opportunities. This is possible because animals will often increase investment in reproduction as their prospects for survival decrease, such as when immune‐challenged (i.e., terminal investment sensu Clutton‐Brock, 1984). That sexually attractive male *G. texensis* crickets increase their calling effort when immune‐challenged (Kelly et al., 2015) supports this hypothesis; however, other empirical evidence suggests that reproductive behavior is generally diminished in immune‐challenged crickets (Adamo, Gomez‐Juliano, LeDue, Little, & Sullivan, 2015; Jacot et al., 2004).

Perhaps, sick crickets in our study spent as much time out of shelters as control individuals because they were searching for food to fuel their immune response. This is possible as we did not find any differences in feeding behavior between treatment and control crickets and so sick crickets might have matched the feeding rate of controls by forgoing shelter use. Sullivan et al. (2016) hypothesized that perhaps the immune‐challenged crickets in their study remained outside of shelters to search for particular types of food rather than food in general. They offered this as a possible explanation because insects can alter their food preferences when sick (Ponton et al., 2013) in order to self‐medicate with specific plants (Singer, Mason, & Smilanich, 2014). Although crickets have been shown to avoid lipid‐rich foods when infected (Adamo et al., 2010), we found no evidence of fat‐avoidance in this study.

We did not observe feeding cessation or diet selectivity by sick individuals as predicted. We found that independent of sex or immune treatment, crickets spent similar amounts of time on the protein, fat, and carbohydrate diets. This finding contradicts recent studies on insects in which immune‐challenged individuals were selective in their diet choice. Povey et al. (2009) and Lee et al. (2006) showed that when caterpillars (*Spodoptera exempta* and *S. littoralis*, respectively) were immune‐challenged with *Bacillus subtilis* and nucleopolyhedrovirus, respectively, were allowed to self‐select their diet, they chose to eat diets that were higher in protein presumably because the intake of protein will reduce the protein costs of mounting an immune response. In contrast, Mason, Smilanich, and Singer (2014) showed that immune‐challenged *Grammia incorrupta* caterpillars avoided protein‐rich foods in favor of carbohydrate‐rich ones, which apparently improved melanization responses.

Our data also suggest that longer durations on diets were achieved by frequent visitation; it is rare that a cricket visits a diet or water once and remains for a long period of time. Kelly (2011) also observed a lack of feeding cessation by females in another orthopteran species, the Wellington tree weta (*Hemideina crassidens*), that were repeatedly immune‐challenged with LPS. However, Sullivan et al. (2016) reported that immune‐challenged *G. texensis* field crickets ate significantly less than control crickets. Although crickets in our study appeared to consume food while on the diet, we cannot rule out the possibility that acquisition rates were not constant among the different diets or among individuals. Thus, some individuals might have consumed more food than another cricket despite being on a diet for the same period of time.

We expected immune‐challenged crickets to lose mass during a trial because sick individuals not only expend considerable energy fighting an immune challenge (e.g., Bashir‐Tanoli & Tinsley, 2014; Jacot et al., 2004; Kelly, 2011) but they will also cease feeding, or at least significantly reduce their acquisition of food. Surprisingly, immune‐challenged crickets in our study did not lose mass during trials. Our trials were 3 hr in duration, which might have been too small of a widow to register mass loss, particularly if crickets continued to feed. For example, Jacot et al. (2004) found that LPS‐administered crickets lost significant body mass compared with controls, but this loss was recorded three days after injection. However, Shoemaker and Adamo (2007), in contrast, also did not observe a significant loss of body mass in female crickets 14 d after immune system activation. Perhaps mass loss in crickets is best observed a few days after injections rather than within hours or after two weeks.

Taken together our results show little support for sickness behavior in crickets. Our results are largely consistent with studies on another cricket species (Sullivan et al., 2016) but not with respect to other insects (e.g., Bos et al., 2012; Kazlauskas et al., 2016). Sullivan et al. (2016) noted that a lack of sickness behavior in a cricket is in line with the hypothesis that because ectotherms lack physiological fever there is little adaptive value in them conserving energy by reducing activity or increasing shelter use as observed in endotherms. Further studies within a nutritional geometric framework (Raubenheimer & Simpson, 2009) should elucidate whether sick crickets shift their dietary preferences to facilitate immunological responses.

## CONFLICT OF INTEREST

The authors declare that they have no competing interests.

## AUTHOR CONTRIBUTION

**Clint D. Kelly:** Conceptualization (lead); Data curation (equal); Formal analysis (lead); Funding acquisition (lead); Investigation (equal); Methodology (equal); Project administration (equal); Resources (equal); Software (lead); Supervision (lead); Validation (lead); Visualization (lead); Writing‐original draft (lead); Writing‐review & editing (supporting). **Jules Mc Cabe Leroux:** Conceptualization (supporting); Data curation (equal); Formal analysis (supporting); Funding acquisition (supporting); Investigation (equal); Methodology (equal); Project administration (supporting); Resources (supporting); Software (supporting); Supervision (supporting); Validation (supporting); Visualization (supporting); Writing‐original draft (supporting); Writing‐review & editing (equal).

## Data Availability

Data and code are archived and publicly accessible on the Open Science Framework (https://doi.org/10.17605/OSF.IO/H278P).
